# Supplementary material for: Complexome profiling of the Chlamydomonas psb28 mutant reveals TEF5 as an early PSII assembly factor
Source: Plant Cell. 2025 Mar 17;37(6):koaf055. doi: 10.1093/plcell/koaf055 (PMC12164590; doi:10.1093/plcell/koaf055)
Supplement: koaf055_Supplementary_Data [file koaf055_supplementary_data.zip › psb28_interactive.zip!/plots/intensity/qualityPlots/peptides/_Cre09.g401350.t1.2.html]

### Cre09.g401350.t1.2

Trivial name:   
SeqCoverage [median]: 13.90  
Mass: 25.28 kDa  
  
 

| Sequence | MissedCleavage | Mass [Da] | wtIntensity | muIntensity | Score |
| --- | --- | --- | --- | --- | --- |
| LKPENWEANLTAPGTR | 1 | 1795.92 | 0.00 | 1661.80 | 52.03 |
| LVYTVSGK | 0 | 865.49 | 787.97 | 0.00 | 58.92 |
| SGGVALWGR | 0 | 901.48 | 246.65 | 0.00 | 71.50 |
|  |  |  | 1034.6 | 1661.8 |  |
